# In this month’s Bulletin

**DOI:** 10.2471/BLT.19.000119

**Published:** 2019-01-01

In editorials this month, Diana Zandi et al. (2) call for papers on the subject of artificial intelligence in the health sector. Wenjing Tao et al. (3) discuss the proxy indicators used to estimate antibiotic consumption by people and the continued surveillance needed to control antimicrobial resistance.

Tatum Anderson and Gary Humphreys (6–7) report on recent outbreaks and global spread of Zika virus disease. Catalina Devandas Aguilar talks to Stephanie Cheng (8–9) about the impact of the 2008 *Convention on the Rights of Persons with Disabilities*.

## Cameroon, Côte d’Ivoire, Democratic Republic of the Congo, Ethiopia, Kenya, Lesotho, Malawi, Mozambique, Namibia, Nigeria, Rwanda, Uganda, United Republic of Tanzania, Zambia, Zimbabwe

## Removing barriers to access for adolescents

Britt McKinnon & Ashley Vandermorris (42–50) query the associations between age-of-consent to testing and knowledge of HIV status. 

## China

### Vaccinating dogs and people

Yuehong Wei et al. (51–58) analyse decades of rabies control efforts.

## Guatemala, Mexico, Peru and the Plurinational State of Bolivia

### Indigenous languages and disparities

Nancy Armenta Paulino et al. (59–67) examine inequities in the provision of maternal health care. 

## Kenya

### Excluded from household surveys

Paula Braitstein et al. (33–41) measure HIV prevalence in young people and children living on the streets.

## Nigeria 

### Redirecting the polio programme 

Samuel Bawa et al. (24–32) study the feasibility of delivering primary health care instead of polio vaccines. 

## South Africa

### Living with HIV, diabetes, hypertension

Angela Y Chang et al. (10–23) track how people fare when they have other chronic diseases.

